# Genome Sequence of the Autotrophic Acetogen *Clostridium autoethanogenum* JA1-1 Strain DSM 10061, a Producer of Ethanol from Carbon Monoxide

**DOI:** 10.1128/genomeA.00628-13

**Published:** 2013-08-15

**Authors:** José M. Bruno-Barcena, Mari S. Chinn, Amy M. Grunden

**Affiliations:** Department of Plant and Microbial Biology, North Carolina State University, Raleigh, North Carolina, USAa; Department of Biological and Agricultural Engineering, North Carolina State University, Raleigh, North Carolina, USAb

## Abstract

*Clostridium autoethanogenum* is an anaerobic, autotrophic acetogen that is capable of converting CO and CO_2_ into ethanol and acetate. Here we report the draft genome sequence of *C. autoethanogenum* JA1-1 strain DSM 10061 (4.5 Mbp; G+C content, 37.5%) and the findings obtained from annotation of the genome sequence.

## GENOME ANNOUNCEMENT

*Clostridium autoethanogenum* is a Gram-positive, anaerobic, rod-shaped, spore-forming, motile bacterium that was enriched from rabbit feces and isolated using carbon monoxide as the sole source of energy and carbon (1). *C. autoethanogenum* has biotechnological significance because it can use C_1_ gases (CO and CO_2_) that are present in industrial waste gas streams and synthesize gas to generate alcohols and acetate (1–3). Additionally, *C. autoethanogenum* can metabolize sugars derived from cellulose deconstruction, such as xylose, arabinose, fructose, and rhamnose (1).

The Genomic Science Laboratory (GSL) at NC State University and the Microbiome Core Facility at the University of North Carolina, Chapel Hill, generated shotgun sequence data for *C. autoethanogenum* using 454 GS FLX Titanium and Ion Torrent Personal Genome Machine (PGM) techniques. The 454 GS FLX Titanium system generated 452,052 reads with sequence lengths averaging 600 bp, and the Ion Torrent PGM generated 453,686 reads with sequence lengths averaging 260 bp. Preliminary assembly of 905,738 raw reads was performed using Newbler software (Roche). This assembly was manually revised and improved to obtain a quality draft of 100 contigs. The genome sequence was structurally and functionally annotated with the Rapid Annotations using Subsystems Technology (RAST) server (4). A comparison of *C. autoethanogenum* gene-coding sequences with those of *Clostridium ljungdahlii* DSM 13528 was also conducted using this method. A total of 749,699 reads were assembled with Geneious (Biomatters, New Zealand) using a *Clostridium ljungdahlii* DSM 13528 sequence (5) (accession no. CP001666) as the reference sequence. *C. autoethanogenum* JA1-1 strain DSM 10061 has a genome size of 4.5 Mbp, similar to that of *C. ljungdahlii* (4.6 Mbp), and a G + C content of 37.5%. It contains 4,135 predicted coding sequences.

16S rRNA gene-based analysis of the genus *Clostridium* suggested that *C. autoethanogenum* and *C. ljungdahlii* are phylogenetically indistinguishable (6). JSpecies (v 1.2.1) (7) comparison of *C. autoethanogenum* JA1-1 versus *Clostridium ljungdahlii* DSM 13528 provides TETRA correlation values of 0.9977, an average nucleotide identity using BLAST (ANIb) (8) of 99.05% (ANIb aligned, 93.33%), and an average nucleotide identity using MUMmer (ANIm) (9) of 98.82% (ANIm aligned, 93.82%). Therefore, these two organisms share a large number of genes, and the TETRA/ANIm values of 0.9977/98.82 suggest that they can be taxonomically classified as the same species. However, phenotypic differences suggest they are different strains. Differences in cell density for *C. ljungdahlii* and *C. autoethanogenum* (472 mg [dry weight] cells/liter/optical density at 600 nm [OD_600_] and 317 mg [dry weight] cells/liter/OD_600_, respectively) are observed under similar growth conditions. The rate and extent of carbon utilization are lower in *C. autoethanogenum* (3, 10). Unlike *C. ljungdahlii*, which consistently consumes fructose (5 g/liter, preferred sugar) within 24 h, *C. autoethanogenum* consumes only 3 g/liter of xylose (preferred sugar) over 48 h (10). *C. ljungdahlii* has demonstrated an enhanced metabolic capacity to use syngas with improved bioenergetics when pre-adapted to fructose (11), whereas exposure of *C. autoethanogenum* to its preferred sugar does not. These phenotypic distinctions are likely related to discrete differences in the sequences of the *Clostridium* strains.

### Nucleotide sequence accession numbers.

This whole-genome shotgun project has been deposited at DDBJ/EMBL/GenBank under the accession number ASZX00000000. The version described in this paper is version ASZX01000000.
